# Lifetime incidence and age of onset of mental disorders, and 12-month service utilization in primary and secondary care: a Finnish nationwide registry study

**DOI:** 10.1017/S2045796025100061

**Published:** 2025-06-06

**Authors:** Kimmo Suokas, Ripsa Niemi, Mai Gutvilig, John J. McGrath, Kaisla Komulainen, Jaana Suvisaari, Marko Elovainio, Sonja Lumme, Sami Pirkola, Christian Hakulinen

**Affiliations:** 1 Department of Psychology, Faculty of Medicine, University of Helsinki, Helsinki, Finland; 2 Faculty of Social Sciences, Tampere University, Tampere, Finland; 3 Queensland Centre for Mental Health Research, University of Queensland, Brisbane, QLD, Australia; 4 Finnish Institute for Health and Welfare, Helsinki, Finland; 5 Department of Psychiatry, The Pirkanmaa Wellbeing Services County, Tampere, Finland

**Keywords:** ADHD, adolescents, bipolar disorder, child psychiatry, epidemiology, psychosis

## Abstract

**Background:**

Previous studies have estimated the lifetime incidence, age of onset and prevalence of mental disorders, but none have used nationwide data covering both primary and secondary care, even though mental disorders are commonly treated in primary care. We aimed to determine lifetime incidence, age-specific incidence, age of onset and service utilization for diagnosed mental disorders.

**Methods:**

This register-based cohort study followed the entire population of Finland from 2000 to 2020. We estimated the cumulative incidence of diagnosed mental disorders with the Aalen–Johansen estimator, accounting for competing risks such as death and emigration. We also calculated age-specific incidence and 12-month service utilization as of 31 December 2019, providing diagnosis-, age- and gender-specific estimates.

**Results:**

We followed 6.4 million individuals for 98.5 million person-years. By age 100, lifetime incidence of any diagnosed mental disorder was 76.7% (95% CI, 76.6–76.7) in women and 69.7% (69.6–69.8) in men; in psychiatric secondary care, it was 39.7% (39.6–39.8) and 31.5% (31.4–31.6). At age 75, stricter estimates for non-organic disorders (ICD-10: F10–F99) were 65.6% (65.5–65.7) for women and 60.0% (59.9–60.1). Anxiety disorders (F40–F48) had the highest cumulative incidence. Median age of onset of non-organic mental disorders was 24.1 (interquartile range, 14.8–43.3 years) in women and 20.0 (interquartile range, 7.3–42.2 years) in men. Service utilization within 12 months was 9.0% for women and 7.7% for men.

**Conclusions:**

Most, though not all, individuals experience at least one type of mental disorder, often during youth. Capturing the overall occurrence of mental disorders requires including both primary and secondary care data.

## Introduction

Mental disorders are prevalent, commonly have their first onset in childhood and adolescence, tend to shift from one to another and thus constitute a major source of years lived with disability throughout the life course (Caspi *et al.*, 2020; Kessing *et al.*, 2023; Kieling *et al.*, 2024; McGrath *et al.*, 2023; Yang *et al.*, 2024). Understanding the fundamental aspects of lifetime incidence, age of onset and service utilization for different mental disorders may help conceptualize mental disorders, identify windows for interventions and plan efficient services.

Recent findings indicate that mental disorders eventually affect almost everyone. A birth cohort from New Zealand reported a cumulative incidence of 86% by the age of 45 (Caspi *et al.*, 2020). In a Danish register-based study combining data on secondary care and psychotropic medication prescriptions (as a proxy marker for a diagnosis of mental disorders), the lifetime cumulative incidence of a mental disorder was 82.6% by the age of 100 (Kessing *et al.*, 2023). On the other hand, major survey studies have estimated that approximately half of the population experience a mental disorder by the age of 75 years (Kessler *et al.*, 2005; McGrath *et al.*, 2023). Even though mental disorders are commonly treated in primary care (Caspi *et al.*, 2024; Suokas *et al.*, 2022), data on treatments from secondary care are often generalized to all mental disorders. This may lead to a biased understanding of the epidemiology of mental health conditions at the population level. Regarding variability in lifetime risk estimates of mental disorders, it is important to note that no comprehensive estimates exist for the lifetime incidence of all diagnosed mental disorders across all treated conditions.

Previous studies on lifetime incidence differ regarding the age considered – 75, 80 or 100 years – and whether organic and other neuropsychiatric diagnoses were included, introducing another source of variation in the reported lifetime estimates (Beck *et al.*, 2024; McGrath *et al.*, 2023; Pedersen *et al.*, 2014). In Finland, where the median age at death is currently 85 for women and 78 for men, it has been estimated that over 20% of women born after 1975 will live beyond 100 years, indicating the relevance of cumulative incidence estimates at various ages (Myrskylä, 2010; Statistics Finland, 2023). To date, there are no nationwide estimates of cumulative incidence of all diagnosed mental disorders in both primary and secondary care at all ages.

Similarly, several studies have evaluated the age of onset of mental disorders, but comprehensive nationwide reports are lacking so far. Based on a meta-analysis of survey studies, incidence peaks at the age of 14.5 years (Solmi *et al.*, 2022). There is substantial variation in the peak age of onset by gender and diagnosis, with the traditional childhood-onset disorders showing the earliest age of onset and organic mental disorders the latest (Beck *et al.*, 2024; Dalsgaard *et al.*, 2020; McGrath *et al.*, 2023; Pedersen *et al.*, 2014; Solmi *et al.*, 2022).

Mental disorders cause remarkable burden throughout life with varying patterns of remission and relapse (GBD 2019 Mental Disorders Collaborators, 2022; Solmi *et al.*, 2023). While estimates of the prevalence of mental disorders vary between studies (Barican *et al.*, 2022; Ten Have *et al.*, 2023), age- and diagnosis-specific analyses of medical service utilization provide an indirect measure of both incident and chronic or recurrent cases in clinical settings. Such analyses may help estimate the overall need for care across the life course.

The aims of the present study were to provide a more detailed characterization of how common mental disorders are by estimating lifetime and age-specific cumulative incidence, the age of onset and 12-month age-specific overall and diagnosis-specific service utilization for diagnosed mental disorders. For the first time, these estimates are based on nationwide population-based register data, covering both primary and secondary care.

## Method

This register-based cohort study included all individuals born in Finland or elsewhere, from 1 January 1900 through 31 December 2019, and present in the Finnish population register at some point between 1 January 2000 and 31 December 2020.

The Research Ethics Committee of the Finnish Institute for Health and Welfare approved the study protocol (decision #10/2016§751). Data were linked with permission from Statistics Finland (TK–53–1696-16) and the Finnish Institute of Health and Welfare. Informed consent is not required for register-based studies in Finland.

### Data sources

Data on the time of birth, death and permanent emigration from Finland were extracted from Statistics Finland’s population register, covering the total population on the last day of each study year.

Information on healthcare contacts was obtained from the Finnish Care Register for Health Care, which show good consistence and adequate diagnostic reliability (Sund, 2012) and the Register of Primary Health Care Visits. Psychiatric inpatient care can be dependably recognized since 1975, secondary outpatient care has been included since 1998 and primary care since 2011 (Suokas *et al.*, 2024a).

The *International Statistical Classification of Diseases and Related Health Problems, Tenth Revision* (*ICD–10*) has been used in Finland since 1996. Prior to that, the Finnish version of the ICD–9 was used from 1987 to 1995, and ICD–8 from 1969 to 1986. In some primary care facilities, the International Classification of Primary Care, Second Edition (ICPC–2), is used instead of ICD–10. These diagnoses were converted to corresponding ICD–10 sub-chapter categories, and the registers were preprocessed for maximizing the accuracy of the data (Suokas *et al.*, 2024a).

### Study design

The primary estimate of incident mental disorders included diagnoses in inpatient, outpatient secondary services or primary care. We also examined diagnosis-specific incidence and service utilization for ICD–10 sub-chapter categories and various particular diagnostic categories. Persons were followed from 1 January 2000, or the earliest possible age for each disorder (35 years for organic mental disorders, 1 year for commonly childhood-onset disorders and 5 years for others, Table S1), whichever came later. Follow-up ended at the first recorded diagnosis, 100th birthday, death, permanent emigration or 31 December 2020, whichever came first. We excluded disorder-specific prevalent cases at the start of the follow-up period, which included those with inpatient treatments between 1975 and 1999, and those with outpatient care between 1998 and 1999.

Individuals aged under 100 with at least one mental disorder diagnosis in 2019 were identified to calculate the 12-month service utilization rate. This included both first-time and prevalent cases. The denominator included all individuals under 100 in the Finnish population register as of 31 December 2019. The year 2019 was chosen as the most recent pre-COVID-19 data available.

### Statistical analysis

Cumulative incidence estimates the percentage of individuals diagnosed with a mental disorder by a certain age using the Aalen–Johansen estimator, from the earliest possible onset age to the 100th birthday. The cumulative incidence at age 100 estimate the lifetime risk. Death or emigration from Finland was considered as competing risks. The cumulative incidence estimates were calculated for any disorder in the whole population, separately for men and women and separately for specific mental disorders. Gender ratio for the lifetime incidence was calculated.

**Figure 1.**
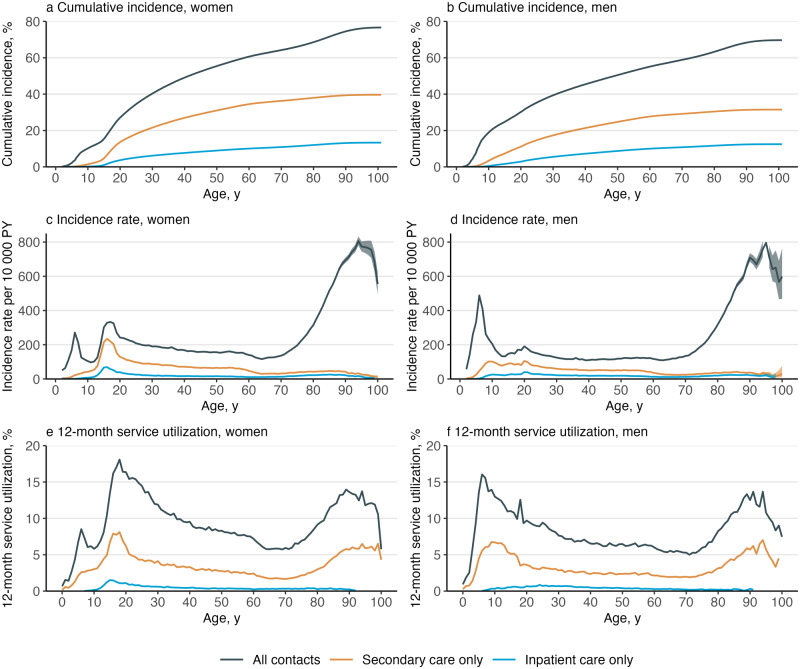
Cumulative incidence, incidence rate and 12-month service utilization^a^ of mental disorders by gender and treatment type.

^a^Service utilization is the number of individuals with any medical contacts with a diagnosis of a mental disorders during the year 2019, divided by the number of individuals in the study population on 31 December 2019.

**Figure 2.**
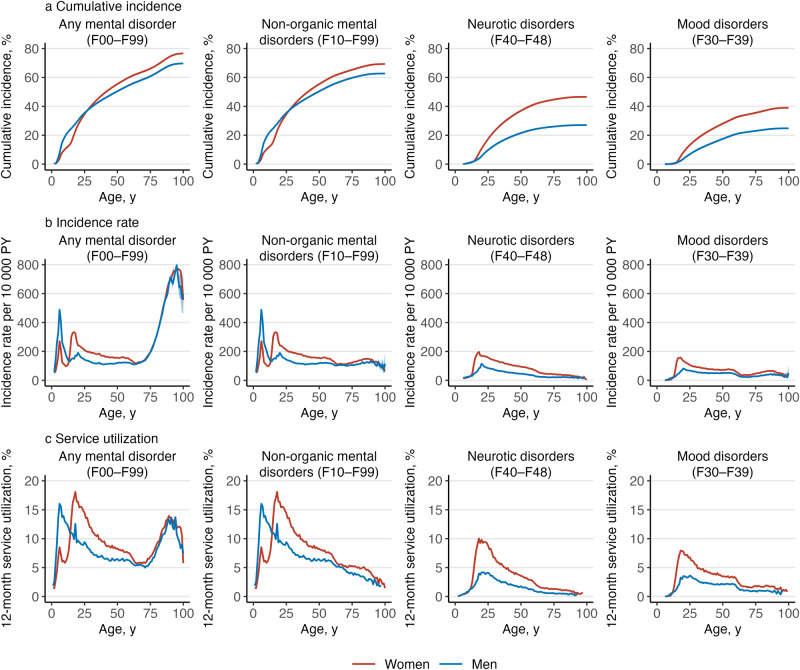
Cumulative incidence, incidence rate and 12-month service utilization^a^ of mental disorders by gender and diagnosis.

^a^Service utilization is the number of individuals with any medical contacts with a diagnosis of a mental disorders during the year 2019, divided by the number of individuals in the study population on 31 December 2019.

**Figure 3.**
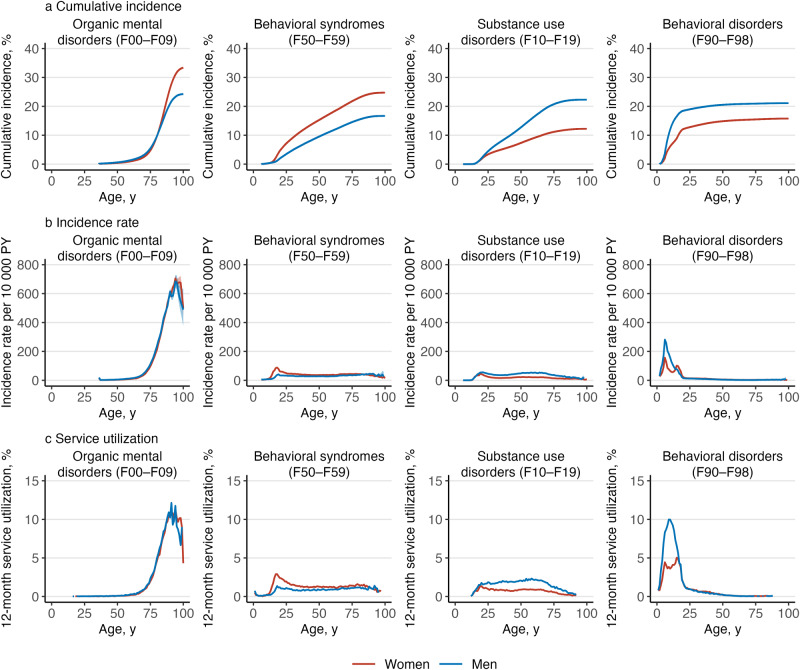
Cumulative incidence, incidence rate and 12-month service utilization^a^ of mental disorders by gender and diagnosis.

^a^Service utilization is the number of individuals with any medical contacts with a diagnosis of a mental disorders during the year 2019, divided by the number of individuals in the study population on 31 December 2019.

**Figure 4.**
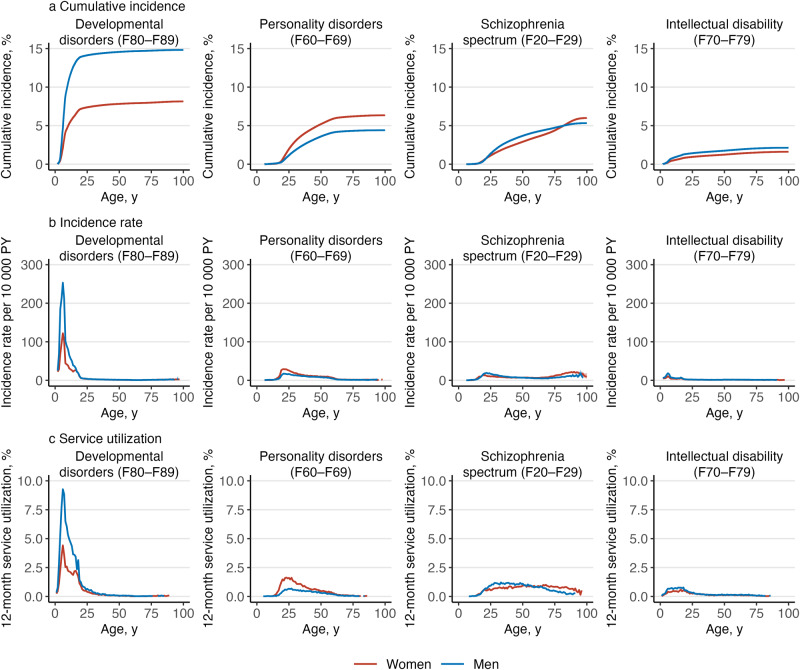
Cumulative incidence, incidence rate and 12-month service utilization^a^ of mental disorders by gender and diagnosis.

^a^Service utilization is the number of individuals with any medical contacts with a diagnosis of a mental disorders during the year 2019, divided by the number of individuals in the study population on 31 December 2019.

Incidence rates across the age range depict the number of people with a first-time mental disorder diagnosis per 10 000 person-years at risk. The incidence rates were estimated at 1-year age intervals to evaluate the most common age to receive a first-time diagnosis. The 95% confidence intervals (CI) were estimated using Poisson regression. Median age of onset was defined as the age at which half of the lifetime incidence was reached (Beck *et al.*, 2024).

Service utilization depicts the percentage of individuals aged under 100 years who had a healthcare visit with a mental disorder diagnosis in the year 2019 and were included in the study population on 31 December 2019. This was estimated in 1-year age intervals and for all ages.

We conducted two sensitivity analyses with stricter criteria for identifying prevalent cases to assess the potential impact of left censoring (due to primary care data being available only since 2011) on the robustness of our lifetime incidence estimates. First, we shortened the study period to 2003–2020, introducing an additional 3-year washout period for inpatient and secondary outpatient treatments and a 1-year retrospective washout for primary care; individuals with any primary care contacts on the year 2011 were excluded at the beginning of follow-up. This excluded individuals with recurrent primary care contacts but introduced potential immortal time and selection bias, as only those at risk of a first primary care contact until 2011 were eligible for exclusion. Second, we restricted follow-up to 2012–2020, with washout periods from 1975 to 2011 for inpatient care, 1998–2011 for secondary outpatient care and 2011 for primary care. This avoided immortal time and selection bias but shortened the study duration. Further details are available in Figure S1. Finally, additional gender ratios and median age of onset estimates were calculated based on cumulative incidence at ages 25, 50 and 75 to evaluate the impact of the definition of lifetime on these estimates.

Analyses were conducted using R version 4.2.2 (The R Foundation).

## Results

Altogether, 6 356 053 Finnish residents were followed for 98.5 million person-years. A total of 1 737 004 persons had their first healthcare contact for any mental disorder during the follow-up, 600 319 persons died, and 157 811 persons were censored due to emigration. Numbers of individuals within each diagnostic category are presented in Table S1. Figure 1 shows the overall cumulative incidence, incidence rate and 12-month service utilization for all mental disorders; Figures 2–4 show the corresponding estimates by ICD–10 sub-chapter category. Results for all specific diagnoses at all specific ages can be seen in interactive online material at https://mentalnet.shinyapps.io/lifetime/.

### Cumulative incidence

Cumulative incidence of any diagnosed mental disorder (ICD–10: F00–F99) at the age 100 years was 76.7% (95% CI, 76.6–76.7) for women and 69.7% (69.6–69.8) for men (Figures 1a and 1b, and Table 1); in secondary care, it was 39.7% (39.6–39.8) for women and 31.5% (31.4–31.6) for men (Figures 1a and 1b, and Table S2); and in psychiatric inpatient care, 13.3% (13.3–13.4) for women and 12.5% (12.4–12.5) for men (Figures 1a and 1b, and Table S3). When organic mental disorders were excluded, cumulative incidence of any diagnosed mental disorder (F10–F99) at the age 100 and 75 years reduced to 69.3% (69.2–69.4) and 65.6% (65.5–65.7) in women and to 62.7% (62.6–62.8) and 60.0% (59.9–60.1) in men, respectively (Figure 2a). Anxiety disorders (F40–F48) showed the highest cumulative incidence in women (46.6% [46.5–46.7]) and in men (27.0% [26.9–27.1]); Table 1 shows the cumulative incidence estimates for each ICD–10 sub-chapter category at different ages. Corresponding estimates for psychiatric secondary care and psychiatric inpatient care alone and a range of more detailed diagnostic categories are shown in Tables S2–S4.

**Table 1.** Cumulative incidence of mental disorders at the ages of 25, 50, 75 and 100 years, and median age of onset (AOO) and interquartile range (IQR) by gender and ICD–10 sub-chapter category

	Cumulative incidence at given ages (95% CI), %										
	Women					Men					
Diagnosis	25	50	75	100	Median AOO (IQR)	25	50	75	100	Median AOO (IQR)	Gender ratio^a^
***Any mental disorder***	35.8 (35.7−35.9)	56.0 (55.9−56.1)	66.6 (66.5−66.6)	76.7 (76.6−76.7)	27.2 (15.5−52.7)	36.3 (36.2−36.4)	51.0 (50.9−51.1)	61.3 (61.2−61.4)	69.7 (69.6−69.8)	23.4 (8.2−52.7)	1.10
Non-organic Mental disorders (F10–F99)	35.8 (35.7−35.9)	56.0 (55.9−56.1)	65.6 (65.5−65.7)	69.3 (69.2−69.4)	24.1 (14.8−43.3)	36.3 (36.2−36.4)	50.9 (50.8−51.0)	60.0 (59.9−60.1)	62.7 (62.6−62.8)	20.0 (7.3−42.2)	1.10
Organic mental disorders (F00–F09)	NA	0.5 (0.4−0.5)	5.5 (5.4−5.5)	33.4 (33.3−33.6)	83.5 (77.9−88.2)	NA	0.7 (0.6−0.7)	6.1 (6.1−6.2)	24.3 (24.2−24.4)	81.1 (74.9−86.2)	1.38
Substance use disorders (F10–F19)	3.6 (3.6−3.7)	7.5 (7.5−7.6)	11.3 (11.2−11.3)	12.2 (12.2−12.3)	41.6 (22.7−59.7)	4.9 (4.8−4.9)	13.1 (13.0−13.2)	20.9 (20.8−21.0)	22.3 (22.2−22.4)	44.5 (26.8−59.9)	0.55
Schizophrenia spectrum (F20–F29)	1.2 (1.2−1.2)	3.0 (2.9−3.0)	4.5 (4.4−4.5)	6.0 (5.9−6.0)	50.5 (28.1−75.4)	1.5 (1.5−1.6)	3.7 (3.7−3.8)	4.8 (4.7−4.8)	5.3 (5.3−5.4)	34.5 (23.8−55.0)	1.13
Mood disorders (F30–F39)	14.1 (14.1−14.2)	28.5 (28.4−28.6)	35.7 (35.6−35.8)	39.0 (38.9−39.1)	32.4 (20.4−51.7)	7.5 (7.4−7.5)	17.8 (17.8−17.9)	23.1 (23.1−23.2)	24.8 (24.7−24.9)	35.4 (22.9−52.2)	1.57
Anxiety disorders (F40–F48)	18.7 (18.6−18.7)	37.5 (37.4−37.6)	44.9 (44.8−45.0)	46.6 (46.5−46.7)	29.3 (19.7−45.1)	10.5 (10.4−10.5)	21.8 (21.7−21.9)	26.1 (26.0−26.2)	27.0 (26.9−27.1)	29.7 (20.3−45.1)	1.72
Behavioural syndromes (F50–F59)	7.6 (7.5−7.7)	15.6 (15.5−15.6)	22.0 (21.9−22.1)	24.7 (24.7−24.8)	38.5 (21.9−61.6)	3.8 (3.7−3.8)	9.8 (9.7−9.8)	14.7 (14.6−14.8)	16.7 (16.6−16.8)	43.5 (26.4−63.7)	1.48
Personality disorders (F60–F69)	2.2 (2.2−2.2)	5.3 (5.2−5.3)	6.2 (6.2−6.3)	6.3 (6.3−6.4)	30.1 (22.5−43.9)	1.3 (1.3−1.3)	3.7 (3.6 − 3.7)	4.3 (4.3 − 4.4)	4.4 (4.4 − 4.4)	32.4 (23.7 − 44.9)	1.44
Intellectual disability (F70–F79)	1.0 (0.9−1.0)	1.3 (1.2−1.3)	1.5 (1.5−1.5)	1.6 (1.6−1.6)	17.0 (7.0−46.1)	1.4 (1.4−1.5)	1.8 (1.7−1.8)	2.1 (2.0−2.1)	2.1 (2.1−2.2)	13.6 (6.0−35.8)	0.75
Disorders of psychological development (F80–F89)	7.4 (7.3−7.5)	7.8 (7.8−7.9)	8.0 (7.9−8.1)	8.1 (8.1−8.2)	6.9 (5.0−13.3)	14.2 (14.1−14.2)	14.6 (14.5−14.7)	14.7 (14.7−14.8)	14.8 (14.7−14.9)	6.2 (4.5−9.9)	0.55
Behavioural and emotional disorders (F90–F98)	12.9 (12.8−13.0)	14.9 (14.8−15.0)	15.5 (15.4−15.6)	15.8 (15.7−15.8)	12.3 (6.3−18.0)	19.1 (19.0−19.2)	20.6 (20.5−20.7)	20.9 (20.9−21.0)	21.1 (21.0−21.2)	8.1 (5.5−12.9)	0.75

a Women-to-men ratio of the cumulative incidence estimates at the age of 100.

Cumulative incidence of any mental disorder was higher in men than in women until the age of 26.2 years, when the value was 37.3% (37.2–37.4) for women and men (Figure 2a). Thereafter, cumulative incidence was higher in women. Behavioural and emotional disorders with onset usually occurring in childhood and adolescence (F90–F98) and disorders of psychological development (F80–F89) were the most common ICD–10 sub-chapter categories in early life; anxiety disorders (F40–F48) became the sub-chapter category with the highest cumulative incidence at the age of 21 in women and 46 in men and remained thereafter (Figure S2).

### Incidence rates and age of onset curves

The age-specific incidence rates in childhood and adolescence showed a bimodal pattern (Figures 1c and 1d). The first peak in incidence was at the age of 6 in both boys and girls with most diagnoses from the sub-chapter categories of disorders of psychological development (F80–F89) and behavioural and emotional disorders (F90–F98) (Figures 3b and 4b). The second peak was at the age of 15–18 in girls and outweighed the first peak, whereas in boys, the second peak was at the age of 20 and was much smaller than the first one. The two most prominent diagnoses at the second peak were mood disorders (F30–F39) and anxiety disorders (F40–F48) in both girls and boys (Figure 2b).

After adolescence, the lowest incidence rates were observed at the age of 64 in women and 39 in men (Figures 1c and 1d). Thereafter, the most incident disorders were dementias (F00–03), but schizophrenia spectrum (F20–F29), mood disorders (F30–F39) and behavioural syndromes (F50–F59) also showed a little increase in incidence rates at late life (Figures 2b, 3b and 4b).

Table 1 shows the gender-specific median age of onset and interquartile range (IQR) for different mental disorders; for non-organic mental disorders (F10–99), the median age of onset was 24.1 years (interquartile range, 14.8–43.3 years) in women and 20.0 years (interquartile range, 7.3–42.2 years) in men.

### 12-month service utilization

Overall, 9.0% of women and 7.7% of men under the age of 100 years had any medical contact with a diagnosis of a mental disorder in 2019 (Table 2). The highest service utilization, 18.1% was observed at the age of 18 in women and 16.0% in men at the age of 6 (Figures 1e and 1f).

**Table 2.** Twelve-month service utilization for medical contacts with diagnosed mental disorders by gender, age group and type of contact in 2019^a^

	Number of individuals and service utilization by age group, *N* (%)				
	0−24	25−49	50−74	75−99	0−99
All contacts					
Women	71 436 (9.9)	86 927 (10.4)	62 322 (6.8)	31 833 (10.0)	252 518 (9.0)
Men	78 841 (10.4)	63 607 (7.2)	50 760 (5.8)	17 739 (8.6)	210 947 (7.7)
Secondary care only					
Women	28 985 (4.0)	28 936 (3.4)	19 626 (2.1)	11 779 (3.7)	89 326 (3.2)
Men	32 647 (4.3)	23 654 (2.7)	18 814 (2.2)	7 164 (3.5)	82 279 (3.0)
Inpatient care only					
Women	4 107 (0.6)	4 139 (0.5)	2 937 (0.3)	958 (0.3)	12 141 (0.4)
Men	2 808 (0.4)	5 059 (0.6)	2 544 (0.3)	403 (0.2)	10 814 (0.4)

a Service utilization is the number of individuals with any medical contacts with a diagnosis of a mental disorders during the year 2019, divided by the number of individuals in the study population on 31 December 2019.

Service utilization diminished throughout adulthood for most ICD–10 sub-chapter categories, with organic mental disorders (F00–F09) being an obvious exception. In addition, schizophrenia spectrum (F20–F29) and substance use disorders (F10–F19) showed relatively stable service utilization in women throughout adulthood. In men, service utilization related to substance use disorders (F10–F19) increased during adulthood, and it was the most commonly present ICD–10 sub-chapter category between ages 58 and 72 (Table S5 and the interactive online material).

### Sensitivity analyses

In the sensitivity analysis with an additional 3-year washout period for inpatient and secondary outpatient treatments and a 1-year retrospective washout for primary care, a lifetime cumulative incidence of 77.7% (77.6–77.8%) in women and 70.9% (70.8–71.0%) in men at age 100 was observed for all disorders. In the second sensitivity analysis with increased washout periods and follow-up restricted to 2012–2020, the corresponding estimates were 86.3% (86.2–86.4%) and 81.0% (80.9–81.1%). The gender ratio in cumulative incidence was sensitive to the choice of age considered as lifetime for organic mental disorders and schizophrenia spectrum disorders (Table S6). The median age of onset for non-organic mental disorders decreased by less than 2 years when lifetime was defined as 75 years (Table S7).

## Discussion

This nationwide cohort study with a 21-year follow-up provides comprehensive estimates of the lifetime cumulative incidence, age of onset and 12-month service utilization for mental disorders across both primary and secondary healthcare services in Finland. Our findings indicate that 77% of women and 70% of men are affected by the age of 100, and 9.0% of women and 7.7% of men have a medical contact with a mental disorder diagnosis within a 12-month period. The highest incidence and service utilization occurred in childhood for boys and in adolescence for girls, with a second peak at around 90 years due to dementia. To our knowledge, this study provides the most extensive analysis of mental disorder incidence and service utilization throughout the life course, with diagnosis-, gender- and age-specific results.

Our estimates of lifetime cumulative incidence are lower than previous Danish estimates (87.5% for women and 76.7% for men), where medication use was used as a proxy for primary care contacts (Kessing *et al.*, 2023). However, in children and youths, our cumulative incidence estimates are higher than those in previous Danish research, likely due to the common use of non-pharmacological treatments in this age group (Dalsgaard *et al.*, 2020; Kessing *et al.*, 2023). The follow-up period in those studies began 5 years earlier than in ours, which also may partially explain the difference. On the other hand, our cumulative incidence estimates at age 75 are higher compared to those previously reported in WHO World Mental Helath Survey data (McGrath *et al.*, 2023). Comprehensive lifetime risk estimates are essential for understanding the nature and impact of mental disorders. For example, excess mortality estimates are lower when all disorders, not just those treated in secondary care, are considered (Suokas *et al.*, 2022).

Schizophrenia-spectrum diagnoses exhibited a relatively high lifetime risk, with incidence persisting throughout the life course and prevalence higher in women, as expected (Van Os *et al.*, 1995). In psychiatric secondary care, current estimates of the lifetime risk for schizophrenia-spectrum disorders are lower than recent estimates from Denmark but higher than those reported approximately a decade ago (Beck *et al.*, 2024; Pedersen *et al.*, 2014). The lifetime risk of narrow schizophrenia (F20) was lower than in both prior Danish studies. Generally, cohort- and register-based studies report higher incidences of psychotic disorders compared to first-contact studies (Hogerzeil *et al.*, 2021).

Findings for specific psychotic disorders should be interpreted with caution. Unspecified psychosis had the highest lifetime incidence among schizophrenia spectrum diagnoses, but capturing specific psychotic disorders often requires specialized algorithms (Sara *et al.*, 2014), which were not applied here. Therefore, some unspecified psychoses may later be reclassified for example as affective or substance use disorders (Suokas *et al.*, 2024b). Nevertheless, unspecified psychosis is the most common diagnosis at discharge after first hospitalization for psychosis in Finland (Holm *et al.*, 2024), reflecting that a category of psychotic disorder without stricter criteria is practical in clinical use.

To our knowledge, this is the first study to analyse the incidence of all mental disorders using nationwide primary and secondary care data. Unlike a recent meta-analysis, which mainly included studies focused on young individuals (Solmi *et al.*, 2022), our data reveal a bimodal pattern with distinct gender differences. In our study, boys showed a prominent peak at age 6, associated with developmental and behavioural disorders, and a smaller peak at age 20. Girls had a smaller peak at age 6 but a strong peak at ages 16–18 due to mood and anxiety disorders. A Danish register study using secondary care data reported similar median age of onset estimates and also found earlier incidence peaks in boys, although the early peak at age 6 was absent, whereas in Sweden the early peak in boys was observed (Beck *et al.*, 2024; Yang *et al.*, 2024). In line with previous research, depression and anxiety were more common and had an earlier onset age in women compared to men. The reasons for this disparity remain unclear (Kirkbride *et al.*, 2024; Salk *et al.*, 2017).

Current 12-month service utilization estimates in young people are a few percentage points smaller than previously reported prevalence estimates (Barican *et al.*, 2022; Castelpietra *et al.*, 2022; Kieling *et al.*, 2024). Medical contacts with diagnoses of developmental and behavioural disorders remain prevalent during childhood and adolescence but decrease sharply by age 25. Similarly, a declining pattern was seen with respect to other diagnoses in young adulthood, suggesting a favourable course for most childhood and adolescent disorders (Patton *et al.*, 2014), except for substance use disorders and schizophrenia spectrum disorders, which showed relatively stable patterns of service utilization through adulthood.

It is important to consider features of the Finnish healthcare system when interpreting our results. Universal health screenings are conducted in child welfare clinics, and school readiness is assessed in preschools before children begin elementary school at age 7. These screenings likely contribute to the observed peaks at age 6. For men, another screening occurs at age 18, before compulsory military or civil service (Appelqvist-Schmidlechner *et al.*, 2011). Finally, the observed incidence and the patterns of service utilization across different age groups also reflect the organization and resources of the healthcare system. For example, some changes in service utilization may be linked to transition ages in mental health services (Reneses *et al.*, 2023). In Finland, specialized services are divided into child, adolescent and adult psychiatry, and the transition from adolescent to adult services around age 20 may increase the risk of dropout.

This study adds comprehensive data to the body of literature showing that most individuals experience a mental disorder at some point of their life, most commonly in childhood and adolescence (Caspi *et al.*, 2020; Kessing *et al.*, 2023; McGrath *et al.*, 2023). The precise definition of a correct diagnostic threshold for mental disorders is a complex question without a clear answer (Clark *et al.*, 2017; Stein *et al.*, 2021; Wakefield, 2016), and in practice, diagnoses may serve various clinical and administrative functions (Bhugra *et al.*, 2017; Herrman *et al.*, 2022; Perkins *et al.*, 2018). This points towards a pragmatic view on the nature of mental disorders in healthcare settings; it is possible that some of the diagnosed disorders might better be conceptualized using the broader term mental health conditions (Reed, 2024; Stein *et al.*, 2024). Furthermore, this study clearly demonstrates the need for diagnostic systems that are usable for primary care practitioners.

The main strength of this study is its inclusion of both primary and secondary care data, because mental disorders are commonly treated in primary care (Caspi *et al.*, 2024; Suokas *et al.*, 2022). For example, attention deficit hyperactivity disorder diagnostics in Finland have largely shifted from specialized psychiatric units to general practitioners (Auro *et al.*, 2024). Universal access to publicly funded care and well-trained general practitioners likely ensures most clinically significant disorders are captured in lifetime estimates. Furthermore, we provided estimates with and without organic mental disorders, as well as diagnosis-specific estimates up to age 100.

This study also has limitations. Ideally, individuals would be followed from birth to death to assess lifetime risk, but such data were unavailable, leaving some uncertainty. Competing risk handling varies across studies; we treated emigration as such, estimating diagnosis risk specific to Finland. To recognize incident cases, a washout period up to 25 years was utilized. Hence, some prevalent disorders were likely missed, particularly among older individuals. Sensitivity analyses suggested this misclassification did not substantially inflate cumulative incidence. Analyses restricted to recent years resulted in higher estimates, probably reflecting increased treatments among youth (Gyllenberg *et al.*, 2018; Kiviruusu *et al.*, 2024), but secular trends were not the focus of this study. Conversely, unmet needs in mental health services are common, and some individuals may delay or avoid care, potentially biasing results. Comorbidity and socioeconomic variation in incidence were not examined. Since 2011, public primary care providers have been required to report to registers, but technical issues and regional differences may affect completeness (THL: Finnish Institute for Health and Welfare, 2025). Private and employer-paid outpatient care, significant in Finland, were not fully captured for the study period. The data included visit dates and clinician-coded diagnoses, but cannot distinguish, for example, between crisis interventions and routine follow-ups. Finally, diagnostic validity has only been systematically assessed before the inclusion of primary care data (Perälä *et al.*, 2007; Sund, 2012; Suokas *et al.*, 2023). Findings may not generalize beyond Nordic settings, due to healthcare and cultural differences.

## Conclusion

This nationwide register study shows that most individuals experience mental disorders at some point in their lives, indicating a high need for mental health services, particularly among young people. High lifetime incidence underscores the overall burden, while age of onset estimates pinpoint key periods for early intervention. Combined with service utilization patterns, these findings may help balance primary and specialized services and optimize resource allocation. They also highlight the importance of carefully selecting source populations in studies of mental disorders to ensure the generalizability of results.

## Supporting information

Suokas et al. supplementary material Suokas et al. supplementary material

## Data Availability

The data that support the findings of this study are available from the National Institute of Health and Welfare (www.thl.fi) and Statistics Finland (www.stat.fi). Restrictions apply to the availability of these data, which were used under license for this study. Inquiries about secure access to data should be directed to data permit authority Findata (findata.fi/en). Code used for analysis is available online at https://github.com/kmmsks/lifetime.

## Supplementary material

The supplementary material for this article can be found at https://doi.org/10.1017/S2045796025100061.
